# Knockdown of *Bmp1* and *Pls1* Virulence Genes by Exogenous Application of RNAi-Inducing dsRNA in *Botrytis cinerea*

**DOI:** 10.3390/ijms24054869

**Published:** 2023-03-02

**Authors:** Maria Spada, Claudio Pugliesi, Marco Fambrini, Diego Palpacelli, Susanna Pecchia

**Affiliations:** 1 Department of Agriculture Food and Environment, University of Pisa, Via del Borghetto 80, 56124 Pisa, Italy; 2 Interdepartmental Research Center Nutrafood “Nutraceuticals and Food for Health”, University of Pisa, Via del Borghetto 80, 56124 Pisa, Italy

**Keywords:** gray mold, lettuce, plant protection, topical application of dsRNA, post-transcriptional gene silencing, RNAi-based fungicides

## Abstract

*Botrytis cinerea* is a pathogen of wide agronomic and scientific importance partly due to its tendency to develop fungicide resistance. Recently, there has been great interest in the use of RNA interference as a control strategy against *B. cinerea*. In order to reduce the possible effects on non-target species, the sequence-dependent nature of RNAi can be used as an advantage to customize the design of dsRNA molecules. We selected two genes related to virulence: *BcBmp1* (a MAP kinase essential for fungal pathogenesis) and *BcPls1* (a tetraspanin related to appressorium penetration). After performing a prediction analysis of small interfering RNAs, dsRNAs of 344 (*BcBmp1*) and 413 (*BcPls1*) nucleotides were synthesized in vitro. We tested the effect of topical applications of dsRNAs, both in vitro by a fungal growth assay in microtiter plates and in vivo on artificially inoculated detached lettuce leaves. In both cases, topical applications of dsRNA led to gene knockdown with a delay in conidial germination for *BcBmp1*, an evident growth retardation for *BcPls1*, and a strong reduction in necrotic lesions on lettuce leaves for both genes. Furthermore, a strongly reduced expression of the *BcBmp1* and *BcPls1* genes was observed in both in vitro and in vivo experiments, suggesting that these genes could be promising targets for the development of RNAi-based fungicides against *B. cinerea*.

## 1. Introduction

*Botrytis cinerea* Persoon: Fries (teleomorph *Botryotinia fuckeliana* (de Bary) Whetzel) is a polyphagous necrotrophic pathogenic fungus that causes significant economic losses to agricultural production. The fungus infects mainly dicotyledonous crop species worldwide and can attack most plant parts such as leaves, flowers, stems, petioles, and fruits [1,2].

Unlike other plant pathogenic fungi, *B. cinerea* has a prominent role year-round and may cause infections in a wide range of climatic conditions. *B. cinerea* is recognized as a “high-risk” pathogen in terms of its resistance development to fungicides (Fungicide Resistance Action Committee, FRAC: https://www.frac.info, accessed on 9 November 2022) [3,4,5,6,7].

The resilience of the pathogen to the natural defense mechanisms of plants and toward many fungicides has pushed research toward new control strategies. Recently, novel approaches based on the use of RNA interference (RNAi) are rising up in the crop protection scenario. RNAi is a process of post-transcriptional gene silencing (PTGS) triggered by double-stranded RNA (dsRNA), small interfering RNA (siRNA), or hairpin RNA (hpRNA), resulting in the specific degradation of target mRNA. In particular, RNAi based on the exogenous application of dsRNA has been reported as a non-genetically modified organism (non-GMO) strategy in plant disease control against some pathogenic fungi that involves targeting specific genes. A nucleotide-sequence-specific dsRNA is applied to plants, and it could represent a potential alternative to conventional fungicides [8,9,10,11].

There is great interest in the use of RNAi as a control strategy against *B. cinerea*. Until now, some genes have been targeted for RNAi studies (e.g., effector genes, cell wall elongation genes, ergosterol and chitinase biosynthesis genes, vesicle trafficking pathway genes, and virulence genes involved in signal transduction or in the secretory pathway) [12,13,14,15,16,17,18].

Due to the sequence-dependent nature of RNAi, dsRNA sequences can be customized to reduce the possible effects on non-target species. One of the possible strategies is to use highly specific genes of the pathogen such as virulence genes, which are much less likely to have adverse effects on non-target organisms. Therefore, in view of the future perspectives on the use of RNAi for the control of *B. cinerea* (SIGS, the in vivo production of dsRNA, and dsRNA-based formulations), the use of this type of gene is much more useful and less risky than the use of essential genes.

The infection process of *B. cinerea* involves stages of conidial attachment, germination, host penetration, primary lesion formation, lesion expansion, and tissue maceration, followed by sporulation [19]. Many studies based on gene inactivation approaches have highlighted virulence genes that are closely linked to the various phases of the *B. cinerea* infection [20]. According to a recent classification [21], the sensu lato virulence genes in *B. cinerea* include: (i) a gene associated with the appressorium formation [22], (ii) sensu stricto virulence genes according to the definition of Choquer et al. [20], and (iii) plant cell wall disassembly genes (CAZyme genes) [23].

In previous studies, to minimize off-target problems, we selected the *B. cinerea* sensu stricto virulence gene *BcBmp3*, which is known to have a key role in the infection process of the pathogen. We demonstrated that our dsRNA that targeted this gene was effective at controlling *B. cinerea* and was highly specific [15]. Moving on, in our RNAi knockdown studies, we selected two other sensu stricto virulence genes: *BcBmp1* (a *Fus3/Kss1*-type MAP kinase essential for fungal pathogenesis) and *BcPls1* (a tetraspanin related to appressorium penetration), which are known in the literature for their use in knockout experiments [20,24].

In eukaryotic organisms, mitogen-activated protein kinases (MAPKs) play critical roles in sensing extracellular signals and regulating various development and differentiation processes. Among MAPKs, the *Fus3/Kss1*-type MAPKs play important roles in pathogenicity in many fungi. Data from earlier studies have shown that the *Fus3/Kss1*-type MAPKs are essential for virulence in fungal pathogens because the knockout mutants for these genes failed to penetrate the host cuticles and/or to grow invasively in host tissues [25]. The *BcBmp1* gene is the ortholog of the yeast *Fus3/Kss1* and of the *Magnaporthe grisea* virulence factor *PMK1* [26].

Gene knockout approaches for the *BcBmp1* gene show that *bmp1* mutants have a reduced growth rate and are non-pathogenic on carnation flowers and tomato leaves due to their inability to penetrate and macerate plant tissues. The *BcBmp1* gene is required for host surface recognition and the penetration ability of germinated conidia in the early infection process. Moreover, the germination of *bmp1* mutant conidia is somewhat retarded when compared to that of the wild type [27,28,29].

Tetraspanins are small eukaryotic integral membrane proteins that are known to have varying functions. In filamentous fungi, three families of tetraspanins (*Pls1*, *Tsp2*, and *Tsp3*) with different distributions among the phyla were identified [30,31]. The most important tetraspanin of ascomycetes seems to be *Pls1*, which was first tetraspanin to be identified as a virulence factor in *Magnaporthe oryzae*. The *Pls1* tetraspanin in *B. cinerea* is necessary for its appressoria-mediated penetration into host plants leaves. Moreover, knockout experiments evidenced that *BcPls1* has an impact on pathogenicity, but is not involved in the germination of asexual conidia or vegetative hyphal fusion events [24,32].

The aim of this study was to assess the effectiveness of an exogenous application of *BcBmp1*- and *BcPls1*-targeting dsRNA molecules in the silencing of *BcBmp1* and *BcPls1* genes in vitro and in vivo on lettuce (*Lactuca sativa* L.) leaves.

We first used dsRNAs in experiments on the pathogenic fungus that investigated mycelial growth, relative gene expression, and conidia germination. Subsequently, artificial inoculation tests with *B. cinerea* were carried out on the detached lettuce leaves where topical dsRNA applications were performed. The knockdown efficacy was evaluated by gene expression and a measurement of necrotic areas. We then verified the effects of dsRNAs on non-target organisms in silico and in vivo. To the best of our knowledge, this is the first report on the use of the sensu stricto virulence genes *BcBmp1* and *BcPls1* in RNAi experiments for the control of gray mold caused by *B. cinerea*.

## 2. Results

### 2.1. In Vitro Silencing of BcBmp1 and BcPls1 Genes Differently Affect Growth and Conidial Germination of B. cinerea

As a first step, we investigated whether a knockdown of the *BcBmp1* and *BcPls1* genes could affect the growth of *B. cinerea* in an axenic culture. Therefore, we generated a 344 bp dsRNA (*BcBmp1*-dsRNA), which was complementary to a portion of the fourth exon of the *BcBmp1* gene (Figure S1A and S2), and a 413 bp dsRNA (*BcPls1*-dsRNA), which was complementary to a portion of the second exon of the *BcPls1* gene (Figure S1B and S3).

**Figure 1:**
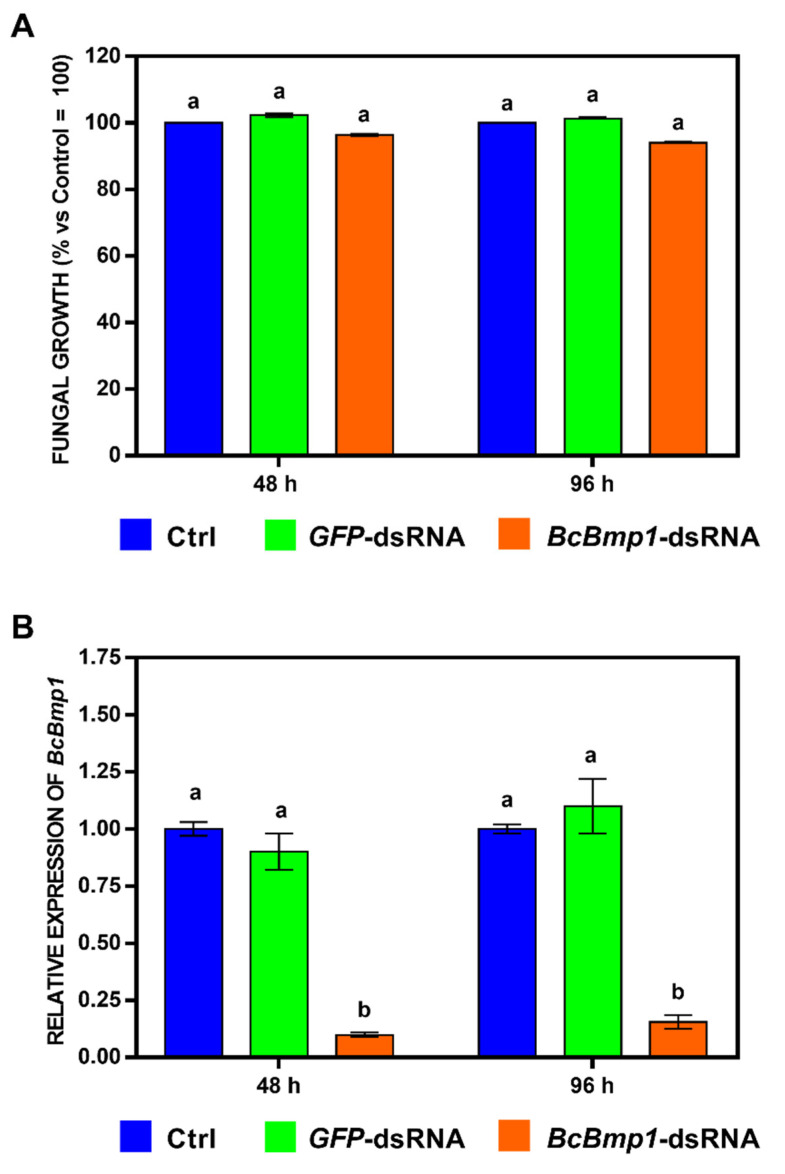
In vitro effects of *BcBmp1*-dsRNA on fungal growth and relative expression levels of the target gene *BcBmp1*. (**A**) Fungal growth was assessed by measuring the optical density (OD_595_) at 48 and 96 h in 96-well microtiter plates. In each well, there were aliquots of a conidial suspension of *B. cinerea* B05.10 (5 × 10^2^ spores) in SMB medium and 2 μg of dsRNA (*GFP*-dsRNA (green) or *BcBmp1*-dsRNA (orange)). SMB + TE buffer was used as control (Ctrl, blue). The graph shows the mean (±SE) of three independent experiments with eight biological replicates (*n* = 8). Same letters above the bars indicate no significant differences from each other (ANOVA) according to Tukey’s HSD test (*p* ≤ 0.05). (**B**) Expression of *BcBmp1* mRNA in mycelium of *B. cinerea* B05.10 treated with *GFP*-dsRNA or *BcBmp1*-dsRNA at 48 and 96 h. SMB + TE buffer was used as control (Ctrl, blue). Relative transcript values were calculated by qRT-PCR using Ctrl and *GFP*-dsRNA as reference samples and were normalized to the *BctubA* gene. The graph shows the mean (±SE) of three biological replicates (*n* = 3). Same letters above the bars indicate no significant differences from each other (ANOVA) according to Tukey’s HSD test (*p* ≤ 0.05). The statistical analysis was conducted separately for 48 and 96 h.

**Figure 2:**
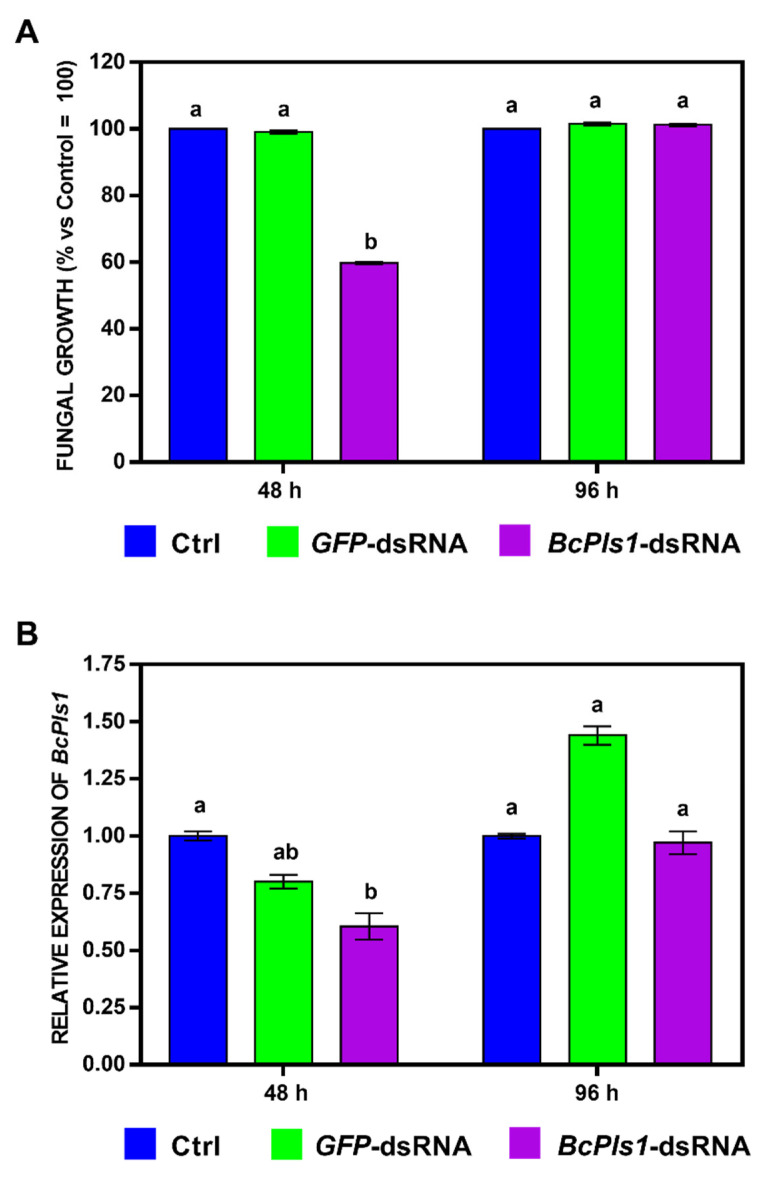
In vitro effects of *BcPls1*-dsRNA on fungal growth and relative expression levels of the target gene *BcPls1*. (**A**) Fungal growth was assessed by measuring the optical density (OD_595_) at 48 and 96 h in 96-well microtiter plates. In each well, there were aliquots of a conidial suspension of *B. cinerea* B05.10 (5 × 10^2^ spores) in SMB medium and 2 μg of dsRNA (*GFP*-dsRNA (green) or *BcPls1*-dsRNA (purple)). SMB + TE buffer was used as control (Ctrl, blue). The graph shows the mean (±SE) of three independent experiments with eight biological replicates (*n* = 8). Same letters above the bars indicate no significant differences from each other (ANOVA) according to Tukey’s HSD test (*p* ≤ 0.05). (**B**) Expression of *BcPls1* mRNA in mycelium of *B. cinerea* B05.10 treated with *GFP*-dsRNA or *BcPls1*-dsRNA at 48 and 96 h. SMB + TE buffer was used as control (Ctrl). Relative transcript values were calculated by qRT-PCR using Ctrl and *GFP*-dsRNA as reference samples and were normalized to the *BctubA* gene. The graph shows the mean (±SE) of three biological replicates (*n* = 3). Same letters above the bars indicate no significant differences from each other (ANOVA) according to Tukey’s HSD test (*p* ≤ 0.05). The statistical analysis was conducted separately for 48 and 96 h.

To quantify the effects of the *BcBmp1*-dsRNA and *BcPls1*-dsRNA on fungal growth, we measured the optical density of the fungal mycelium at different times. For the treatment with *BcBmp1*-dsRNA, the fungal growth was not significantly delayed in the in vitro assay (Figure 1A and Figure S4A). In the case of *BcPls1*-dsRNA, the fungal growth was delayed at 24 (69.8 ± 0.24%), 48 (59.8 ± 0.34%), and 72 h (75.8 ± 0.52%), respectively, compared to the controls (Ctrl = SMB + TE buffer and *GFP*-dsRNA) (Figure 2A and Figure S4B), and was subsequently restored at 96 h (100 ± 0.30%). According to these results, two times 48 and 96 h were chosen to sample the mycelium in order to verify a correlation between reduced fungal growth and the silencing of the target genes.

Quantitative real-time PCR (qRT-PCR) was used to assess the expression of the *BcBmp1* and *BcPls1* genes. After 48 h of incubation in the presence of *BcBmp1*-dsRNA, the expression of the *BcBmp1* gene in the mycelium was significantly suppressed (to about 10%) compared to both control media. Analogously, a reduction in the *BcBmp1* mRNA levels was detected 96 h after treatment (Figure 1B). These data suggest that *BcBmp1*-dsRNA, delivered in vitro, silenced the expression of the *BcBmp1* gene during the vegetative axenic growth of the pathogen.

In contrast, a more complex and diversified *BcPls1* expression was detected. After 48 h of incubation in the presence of *BcPls1*-dsRNA, the expression of the *BcPls1* gene was significantly suppressed (to 60.5%) compared to the Ctrl (Figure 2B). However, the reduction in the *BcPls1* mRNA level was not significant in comparison to the *GFP*-dsRNA-treated mycelium. In addition, at 96 h after treatment, the *BcPls1* expression was not significant different for both Ctrl and *GFP*-dsRNA. These data suggest that the in vitro *BcPls1*-dsRNA was able to silence the expression of the *BcPls1* gene during the early stages of the vegetative axenic growth of the pathogen.

The effects of *BcBmp1*-dsRNA and *BcPls1*-dsRNA on the germination kinetics of *B. cinerea* conidia were determined at 3, 6, and 9 h on liquid SMB in 96-well polystyrene microtiter plates.

**Figure 3:**
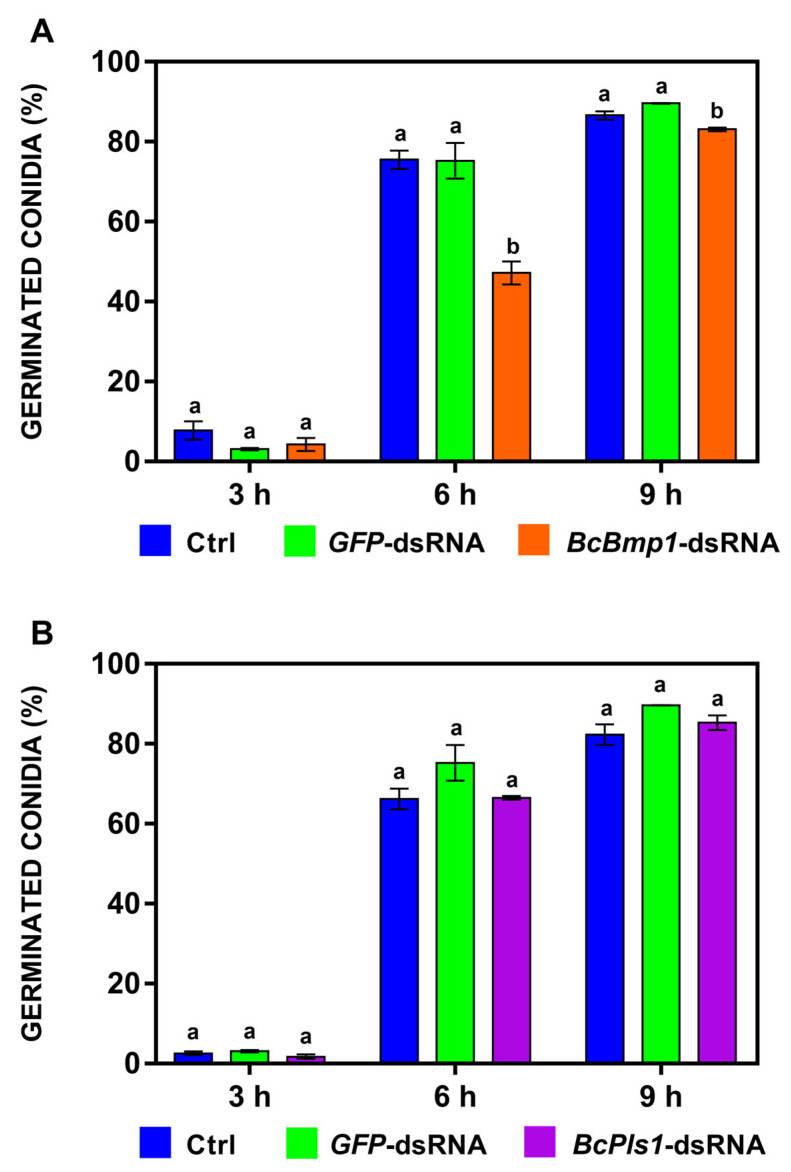
Germination kinetics of *B. cinerea* B05.10 conidia (1 × 10^6^) on liquid SMB in 96-well polystyrene microtiter plates in the presence of (**A**) *BcBmp1*-dsRNA (orange) and (**B**) *BcPls1*-dsRNA (purple). In both graphs, the *GFP*-dsRNA (green) and control (Ctrl = SMB + TE buffer, blue) are reported. The graphs show the mean (±SE) of two independent experiments with three biological replicates (*n* = 3; 200 conidia for each biological replicate were counted). Same letters above the bars indicate no significant differences from each other (ANOVA) according to Tukey’s HSD test (*p* ≤ 0.05). The statistical analysis was conducted separately for 3, 6, and 9 h.

When *BcBmp1*-dsRNA was used, the conidial germination was significantly delayed at 6 and 9 h compared to controls (Ctrl = SMB + TE buffer and *GFP*-dsRNA). On average, the reduction in germination was 37.4% (6 h) and 5.7% (9 h) compared to the germination of both Ctrl and *GFP*-dsRNA. At 3 h, there were no significant differences between the treatments (Figure 3A).

For the treatment with *BcPls1-*dsRNA, the conidial germination was not significantly delayed in the in vitro assay at any of the times tested (Figure 3B).

### 2.2. BcBmp1 and BcPls1 Expression Levels and Size of Necrotic Lesions Are Reduced in Lettuce Leaves Inoculated with Botrytis cinerea and Treated with BcBmp1-dsRNA and BcPls1-dsRNA

We focused on the effects of treatments with *BcBmp1*-dsRNA and *BcPls1*-dsRNA by applying them topically to *Lactuca sativa* cv. Romana. We evaluated the efficacy of dsRNA treatments on the symptoms caused by *B. cinerea* using a detached leaf assay. After a drop application of *BcBmp1*-dsRNA or *BcPls1*-dsRNA (20 ng µL^−1^), the leaves were inoculated with a drop of conidial suspension (5 × 10^2^). Necrotic areas (mm^2^) were recorded and analyzed after 120 h (5 dpi).

**Figure 4:**
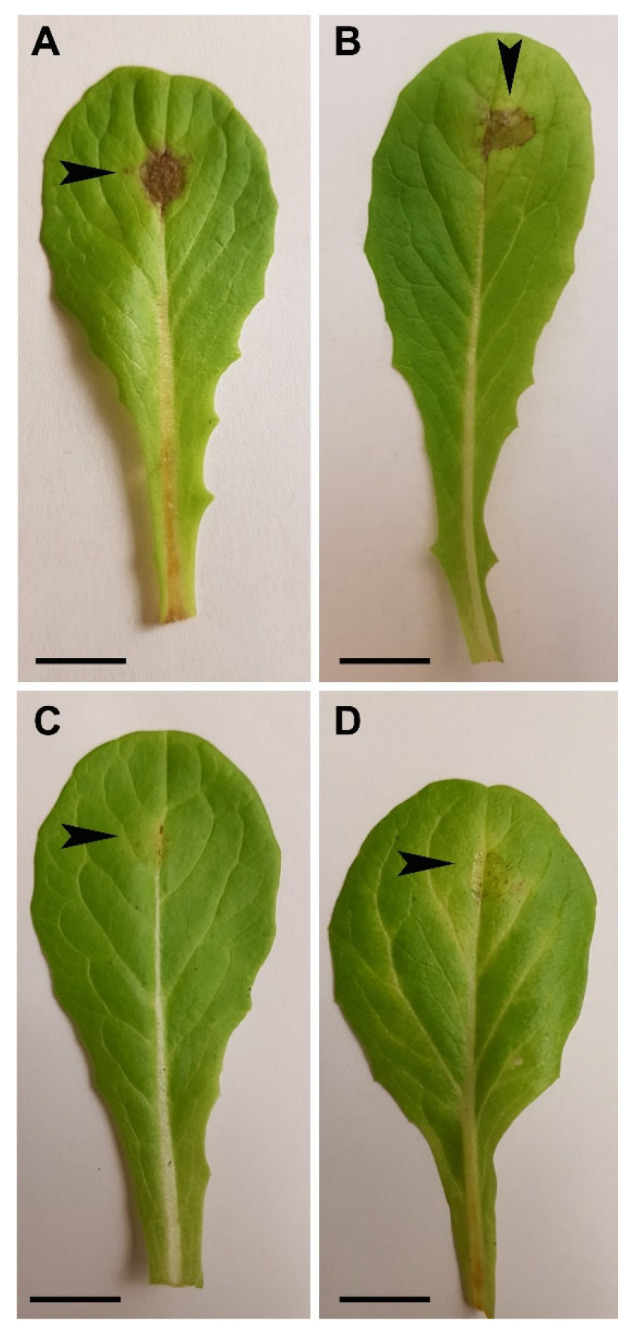
Infection symptoms of *B. cinerea* B05.10 on leaves of *Lactuca sativa* cv. Romana at 5 dpi. (**A**) Leaves were treated with water + TE (Ctrl), (**B**) *GFP*-dsRNA, (**C**) *BcBmp1*-dsRNA, or (**D**) *BcPls1*-dsRNA and were then artificially inoculated with a conidial suspension (5 × 10^2^ spores) of the pathogen. Scale bars = (**A**): 1.19 cm; (**B**): 1.12 cm; (**C**): 1.16 cm; and (**D**): 1.14 cm.

At 5 dpi, the leaves treated with both controls (water + TE buffer and *GFP*-dsRNA at the concentration of 20 ng µL^−1^) showed necrotic lesions, representing a successful *B. cinerea* infection on the lettuce leaves (Figure 4A,B), whereas the *BcBmp1*-dsRNA- or *BcPls1*-dsRNA-treated leaves showed strongly reduced necrotic lesions at the inoculation site (Figure 4C,D).

**Figure 5:**
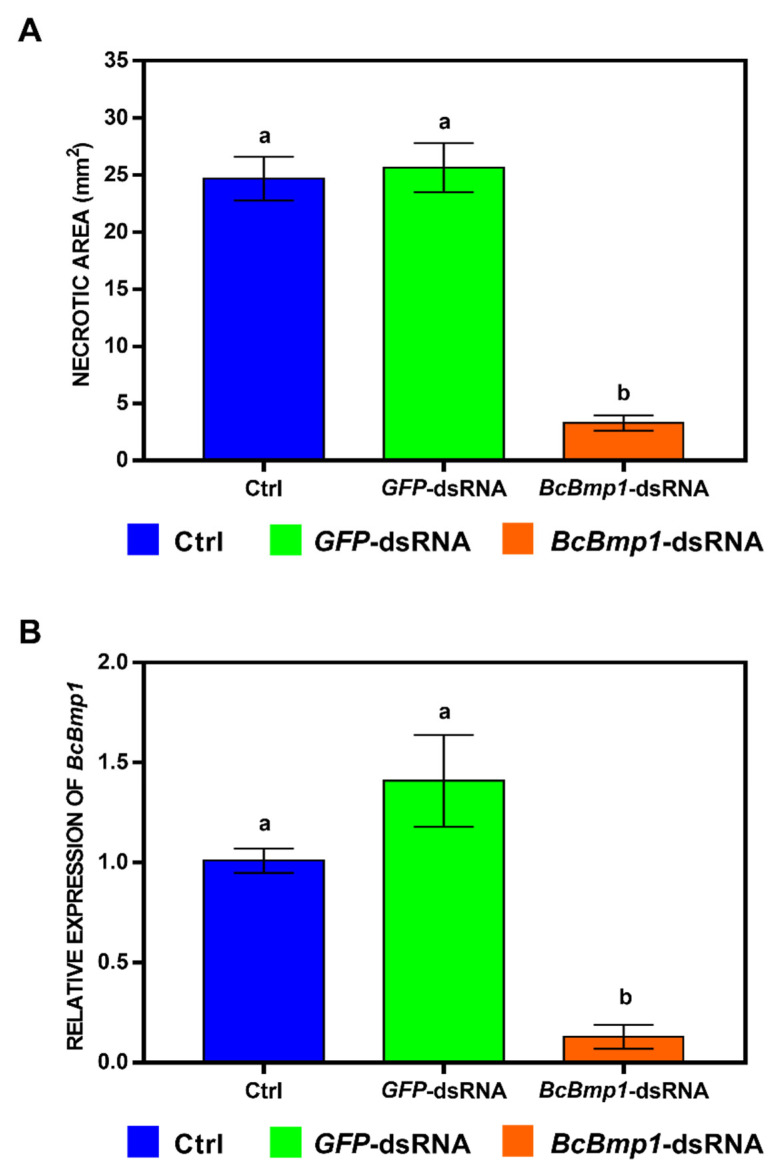
(**A**) Necrotic areas (in mm^2^) caused by *B. cinerea* at 5 dpi, measured using the ImageJ software, version 1.53a. Leaves were treated with water + TE (Ctrl, blue), *GFP*-dsRNA (green), or *BcBmp1*-dsRNA (orange) and were then artificially inoculated with a conidial suspension (5 × 10^2^ spores) of the pathogen. The graph shows the mean (±SE) values of two independent experiments with sixteen biological replicates (*n* = 16). (**B**) Relative transcript values of *BcBmp1* (orange) at 5 dpi were calculated by qRT-PCR using *GFP*-dsRNA (green) and Ctrl (blue) as reference samples and were normalized to the *GAPDH* gene of *Lactuca sativa* (*LsGAPDH*). The graph shows the mean (±SE) values of three biological replicates (*n* = 3). Same letters above the bars indicate no significant differences from each other (ANOVA) according to Tukey’s HSD test (*p* ≤ 0.05).

**Figure 6:**
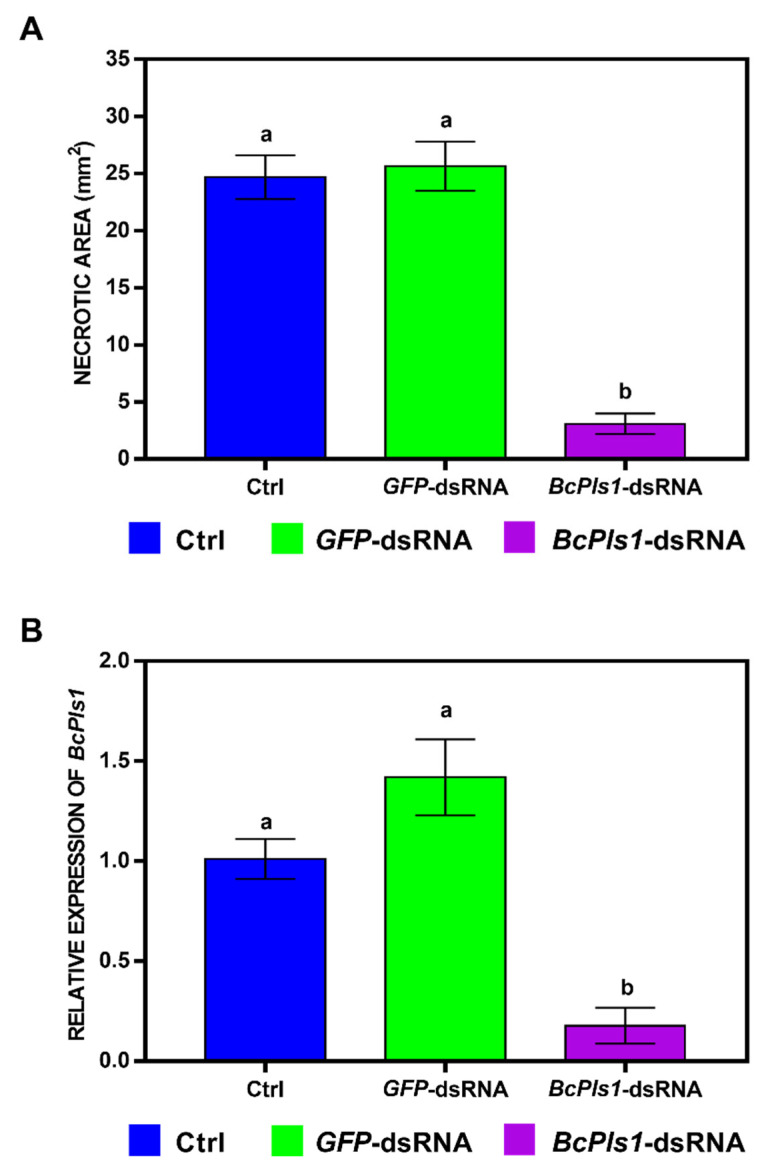
(**A**) Necrotic areas (in mm^2^) caused by *B. cinerea* at 5 dpi measured using the ImageJ software, version 1.53a. Leaves were treated with water + TE (Ctrl, blue), *GFP*-dsRNA (green), or *BcPls1*-dsRNA (purple) and were then artificially inoculated with a conidial suspension (5 × 10^2^ spores) of the pathogen. The graph shows the mean (±SE) values of two independent experiments with sixteen biological replicates (*n* = 16). (**B**) Relative transcript values of *BcPls1* (purple) at 5 dpi were calculated by qRT-PCR using *GFP*-dsRNA (green) and Ctrl (blue) as reference samples and were normalized to the *GAPDH* gene of *Lactuca sativa* (*LsGAPDH*). The graph shows the mean (±SE) values of three biological replicates (*n* = 3). Same letters above the bars indicate no significant differences from each other (ANOVA) according to Tukey’s HSD test (*p* ≤ 0.05).

The lettuce leaves treated with *BcBmp1*-dsRNA (3.3 ± 0.7 mm^2^) or *BcPls1*-dsRNA (3.1 ± 0.9 mm^2^) developed lesions about eight times smaller than those of the control leaves treated with either water + TE buffer (24.7 ± 1.9 mm^2^) or *GFP*-dsRNA (25.6 ± 2.1 mm^2^) (Figure 5A and Figure 6A).

To verify if the reduction at 5 dpi of the *B. cinerea* necrotic areas on both the *BcBmp1*-dsRNA and *BcPls1*-dsRNA-treated leaves was correlated with a knockdown of the genes, qRT-PCR analyses were performed on the inoculated leaves. The *BcBmp1* transcript levels were drastically reduced: 12.8% and 10.6% compared to the Ctrl and *GFP*-dsRNA levels, respectively (Figure 5B). Similarly, the *BcPls1* transcript levels were drastically reduced: 17.6% and 12.5% compared to the Ctrl and *GFP*-dsRNA levels, respectively (Figure 6B).

### 2.3. BcBmp1-dsRNA and BcPls1-dsRNA: Off-Target Prediction and In Vitro Effects against the Off-Target Fungi Trichoderma harzianum T6776 and Fusarium oxysporum DAFE SP21-23

To explore co-silencing effects, we calculated the possible off-target effects for the tested *BcBmp1*-dsRNA and *BcPls1*-dsRNA molecules. Precursor sequences of the constructs were targeted against the complementary DNAs (cDNAs) of different phytopathogenic and beneficial fungi, humans, and lettuce using the si-Fi v21 software (default parameters). The results are summarized in Tables S1 and S2 as the number of siRNA hits found (total and efficient) for each corresponding target.

The prediction of *BcBmp1* off-target transcripts using the si-Fi v21 software evidenced that in one isolate of *Sclerotinia sclerotiorum* alone, 78 siRNAs were found, of which only 37 were efficient (Table S1). The target sequence found (GenBank accession number APA14320) corresponds to the catalytic domain of extracellular signal-regulated kinase 1- and 2-like serine/threonine kinases. This subfamily is composed of the mitogen-activated protein kinases (MAPKs) ERK1, ERK2, baker’s yeast *Fus3*, and similar proteins. The *BcBmp1* gene is the ortholog of the yeast *Fus3/Kss1* and was characterized by gene replacement approaches, but a similar sequence was also found in the genome of *S. sclerotiorum* [22].

In the case of *BcPls1*, the prediction of off-target transcripts using the si-Fi v21 software showed that in *S. sclerotiorum* alone, seven siRNAs were found, of which only four were efficient (Table S2). The target sequences found (GenBank accession numbers EDO03107 and APA12012) corresponded to a hypothetical protein and to a tetraspanin, respectively.

In the in silico analysis of both genes, only a few efficient siRNAs were found in the off-target fungal pathogen *S. sclerotiorum*, which is a close relative of *B. cinerea*. The results obtained by these bioinformatic analyses proved to be a useful guide in the choice of multiple targets for RNAi.

Therefore, the results indicate that *BcBmp1*-dsRNA and *BcPls1*-dsRNA are highly specific for the target *Bmp1* and *Pls1* genes of *B. cinerea*, with no off-target sequences in the host plant (*Lactuca sativa*), distantly related phytopathogenic fungi (*Alternaria alternata*, *Fusarium oxysporum*, *Rhizoctonia solani*, or *Pythium ultimum*), beneficial fungi (*Trichoderma asperellum*, *T. harzianum*, or *Rhizophagous irregularis*), or the human genome. Using the databases of three *B. cinerea* isolates, including B05.10, we found a high number of efficient off-target siRNAs that exactly matched only with the *Bmp1* and the *Pls1* genes, as expected (Tables S1 and S2; Figures S5 and S6).

To further explore the co-silencing effects of the *BcBmp1*-dsRNA and *BcPls1*-dsRNA against off-targets organisms, we tested them against the promising biocontrol agent *T. harzianum* T6776 and against *F. oxysporum* DAFE SP21-23. For these fungi, the off-target prediction analysis with the si-Fi v21 software did not find any efficient siRNAs (Tables S1 and S2).

**Figure 7:**
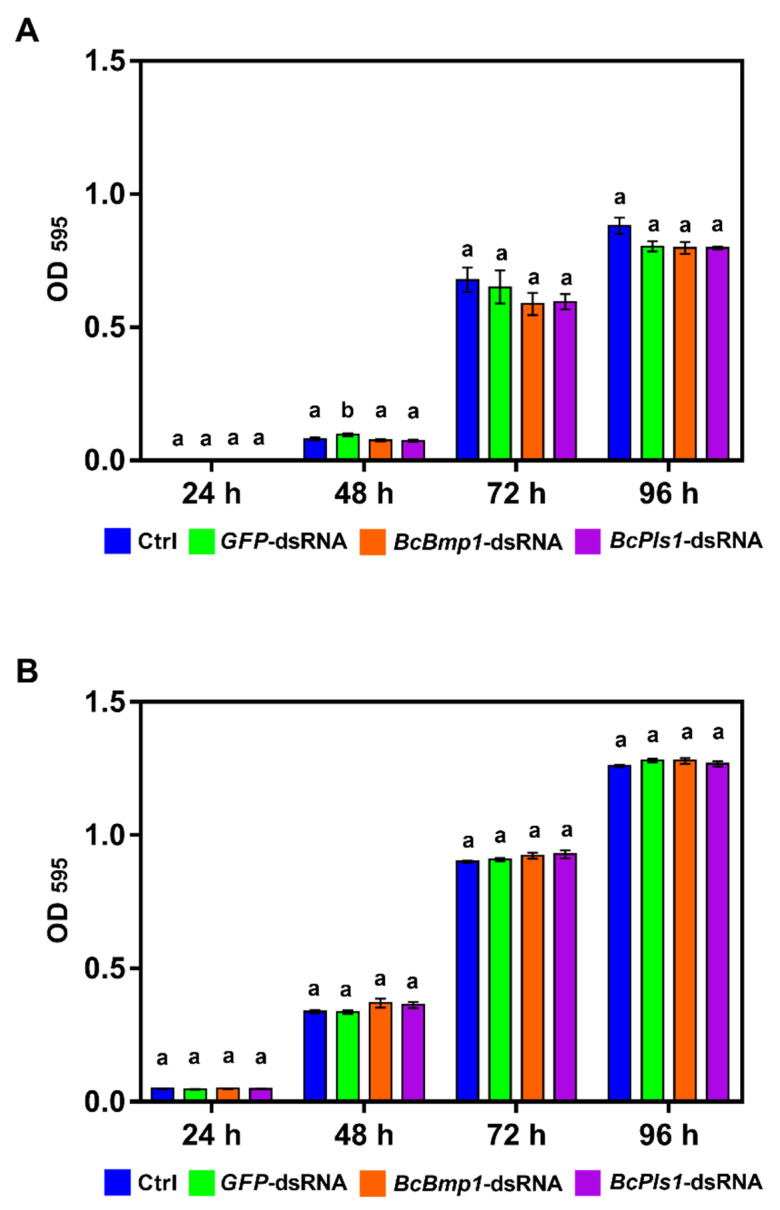
In vitro effects of *BcBmp1*-dsRNA and *BcPls1*-dsRNA on *Trichoderma harzianum* T6776 (**A**) and *Fusarium oxysporum* DAFE SP21-23 (**B**) growth. Fungal growth was assessed by measuring the optical density (OD) at 595 nm at 24, 48, 72, and 96 h in 96-well microtiter plates. In each well, there were aliquots of a conidial suspension of *T. harzianum* T6776 and *F. oxysporum* DAFE SP21-23 (5 × 10^2^ spores) in SMB medium and 2 μg of dsRNA (*GFP*-dsRNA (green) or *BcBmp1*-dsRNA (orange)) or *BcPls1*-dsRNA (purple). SMB + TE buffer was used as control (Ctrl, blue). The graphs show the mean (±SE) values of eight biological replicates (*n* = 8). Same letters above the bars indicate no significant differences from each other (ANOVA) according to Tukey’s HSD test (*p* ≤ 0.05). The statistical analysis was conducted separately for 24, 48, 72, and 96 h.

To quantify the effects of *BcBmp1*-dsRNA and *BcPls1*-dsRNA on fungal growth, the optical density (OD_595_) of the fungal mycelium at different times (24, 48, 72, and 96 h) was measured. The growth of *T. harzianum* T6776 (Figure 7A) and *F. oxysporum* DAFE SP21-23 (Figure 7B) was not significantly reduced at any time compared to the growth of the controls (Ctrl (SMB + TE buffer) and *GFP*-dsRNA), thus confirming both the high specificity of the molecules and the prediction analysis.

## 3. Discussion

In this work, we demonstrated that the *Fus3/Kss1*-type mitogen-activated protein kinase (MAPK) *BcBmp1* and the tetraspanin *BcPls1* genes are efficient and novel targets for RNAi with the purpose of reducing disease symptoms in lettuce after a *B. cinerea* infection. The topical application of *BcBmp1*-dsRNA and *BcPls1*-dsRNA mediated both the in vitro and in vivo knockdown of the *B. cinerea* transcripts. To the best of our knowledge, this is the first report on the reduction of *B. cinerea* virulence through a topical application of dsRNA that targets these sensu stricto virulence genes.

In eukaryotic organisms, mitogen-activated protein kinase (MAPKs) pathways are involved in the transduction of a variety of extracellular signals and in the regulation of many developmental processes [25,26].

Among MAPKs, the yeast and fungal extracellular signal-regulated kinase (YERK1) subfamily is represented by *Fus3/Kss1* in *Saccharomyces cerevisiae*. Different gene knockout studies have shown that *Fus3/Kss1*-type MAPKs are essential for virulence in fungal pathogens because of the failure of phytopathogenic and entomopathogenic fungi knockout mutants to penetrate the host cuticles and/or to grow invasively in host tissues [25,33].

*BcBmp1* is a single-copy gene, and is the ortholog of the yeast *Fus3/Kss1* and of the *Magnaporthe grisea* virulence factor *PMK1*. *bmp1* mutants showed an altered vegetative growth phenotype; the conidia germinated on plant surfaces, but were unable to penetrate and macerate the plant tissues. This clearly indicates that the gene is essential for plant infection; in fact, the MAP kinase pathway seems to be widely conserved in pathogenic fungi and involved in regulating infection (appressorium formation and virulence) processes [27].

Fungal tetraspanins have a key role in the infection process in several pathogenic fungi [34]. The first fungal tetraspanin, *Pls1*, was found in the plant pathogenic fungus *Magnaporthe grisea* (*MgPls1*) [35]. Three fungal tetraspanins homologous to *Pls1* were further identified in the ascomycetes *B. cinerea* (*BcPls1*), *Colletotrichum lindemuthianum* (*ClPls1*), and *Neurospora crassa* (*NcPls1*), defining a novel class of tetraspanins in fungi [36]. Thanks to the increasing availability of fungal genomes in databases, orthologous genes to *Pls1* have been identified in other species of Ascomycetes and Basidiomycetes, but only a single *Pls1* tetraspanin-encoding gene was found in Ascomycetes, facilitating their functional study.

The roles of the tetraspanins *MgPls1*, *BcPls1*, and *ClPls1* were analyzed by inactivating genes through insertional mutagenesis or targeted gene replacement. The corresponding null mutants were unable to infect their host plants, indicating that *Pls1* is essential for appressorium-mediated penetration into the host plant [32,35,37].

For the reasons described above, we considered the sensu stricto virulence genes *Bmp1* and *Pls1* of *B. cinerea* as good potential targets for RNAi mediated by the exogenous application of complementary dsRNAs.

Moreover, the target sequences were chosen so that, in silico, they share no homology with the genes of the host or other off-target organisms. Using the si-Fi v21 software [38], the *BcBmp1*-dsRNA and *BcPls1*-dsRNA were determined to be highly specific for *B. cinerea*, with no off-target hits in the host plant, in distantly related phytopathogenic fungi, in beneficial fungi, or in the human genome. The siRNAs were found to be efficient only in the close relative *Sclerotinia sclerotiorum* (37 for *BcBmp1* and 4 for *BcPls1*).

The choice of highly specific target sequences should avoid homology with off-target transcripts and reduce off-target impacts. Specific software and databases were used to test different regions of a gene in order to minimize off-target hits [39]. Nevertheless, the best approach to reduce risks is to combine bioinformatics analyses with biological data [40].

In light of these considerations, we tested the effects of *BcBmp1*-dsRNA and *BcPls1*-dsRNA molecules against the promising biocontrol agent *T. harzianum* T6776 and the *F. oxysporum* isolate DAFE SP-21-23. Neither of the dsRNAs gave negative results, neither in silico nor in vivo, but their high specificity was confirmed in accordance with the prediction analysis.

In this work, to validate the virulence genes chosen, a first approach was to challenge the fungal pathogen in vitro with the dsRNA molecules. The *BcBmp1*-dsRNA and *BcPls1*-dsRNA molecules were applied to liquid cultures of *B. cinerea*, which was grown in 96-well microtiter plates. This assay is considered very simple, cost-effective, and rapid for quantifying the inhibitory effects of molecules on fungal growth [41]. Furthermore, the assay can be very useful for a preliminary evaluation of the effects of dsRNAs on the vegetative growth and conidial germination of fungi using small amounts of dsRNA [39,42]. The validity of in vitro studies using dsRNA molecules against fungal pathogens has been demonstrated by different studies. The molecules were designed to target essential genes in *Fusarium oxysporum* f.sp. *cubense* and *Mycosphaerella fjiensis* [43], *Sclerotinia sclerotiorum* [13], *F. graminearum* [42], *F. asiaticum* [44], and *B. cinerea* [15,18].

The applications of *BcBmp1*-dsRNA did not affect mycelial growth, but resulted in a delay in conidial germination. Conversely, the applications of *BcPls1*-dsRNA led to a gene knockdown with an evident growth retardation, without significant effects on conidial germination. No alteration in the morphology of the germ tubes was observed in either treatment.

It has been reported that the conidial germination of a *B. cinerea* knockout mutant in the *Bmp1* gene is somewhat retarded when compared to the wild type. In addition, the germ tubes continue to elongate on hydrophobic surfaces and never differentiate into appressoria, indicating that they are defective in the signaling related to appressorium formation [27,28,29].

By comparing the expression data with the vegetative growth data, we highlighted that the strength of the *BcBmp1* gene knockdown is not directly related to the growth retardation, according to other studies [42]. The transcript levels at 48 h were significantly lower than the controls when using a topical application of *BcBmp1*-dsRNA. Gene expression silencing was also observed at 96 h when the vegetative growth was restored. In *S. sclerotiorum*, 48 h were required for optimal RNAi silencing using a topical application of dsRNA, and the level of suppression persisted at 96 h without changing significantly after 48 h [13].

In the case of *BcPls1*-dsRNA, the fungal growth was delayed at 24, 48, and 72 h, respectively, compared to the controls and was subsequently restored at 96 h. Regardless, the expression data at 48 and 96 h suggested that the in vitro *BcPls1*-dsRNA was able to silence the expression of the *BcPls1* gene during the early stages of the vegetative axenic growth of the pathogen.

The inactivation of *BcPls1* in *B. cinerea* by insertional mutagenesis was observed to have no effect on the mycelial growth, conidiation, and conidial germination rate in the *Bcpls1::bar* null mutant. *BcPls1* expression (monitored with *GFP*) is limited to the penetration process in the early stages of infection and is independent of plant signals, since it has also been observed in germinating conidia, germ tubes, and appressoria developed on artificial surfaces [32]. Under our experimental conditions, it was not possible to perform expression studies in the early stages of fungal growth (less than 48 h), as the obtained biomass was too small to yield a sufficient amount of RNA. Furthermore, the observations reported by Gourgues et al. [36] were conducted using a methodological approach that was different from the one used in this work (null mutant vs. RNA silencing mediated by dsRNA).

Gray mold caused by *B. cinerea* is considered one of the main diseases in greenhouse-grown lettuce. The romaine lettuce variety and some iceberg lettuces are susceptible to *B. cinerea*, both in greenhouses and in the field [45].

A second approach used in this work was to evaluate the efficacy of the *BcBmp1*-dsRNA and *BcPls1*-dsRNA molecules at reducing disease symptoms using *Lactuca sativa* cv. Romana-*B. cinerea* as a pathosystem. We performed a detached leaf assay by applying a conidial suspension of the pathogen as the inoculum after locally treating the lettuce leaves with dsRNAs.

A topical application of the *BcBmp1*-dsRNA and *BcPls1*-dsRNA molecules reduced the lesion areas at 5 dpi by approximately eight-fold compared to the controls. This strong decrease in the necrotic areas was associated with a drastically reduced level of *Bmp1* and *Pls1* transcripts on the infected leaves.

Similarly, an external application of dsRNAs targeting different *B. cinerea* genes on the surfaces of fruits, vegetables, and flower petals significantly inhibited gray mold disease [12,13,14,15,16]. Recently, it was demonstrated that a SIGS application of dsRNAs can confer protection for grapevines against *B. cinerea* under both pre- and post-harvest conditions [14,17,18].

Data from many previous studies have shown that *Fus3/Kss1*-type MAPKs are essential for virulence in fungal pathogens due to the failure of pathogen knockout mutants to penetrate the host cuticles and/or to grow invasively in host tissues. Indeed, in *B. cinerea*, *Bmp1* gene-replacement mutants are non-pathogenic on carnation flowers and tomato leaves due to their inability to penetrate and macerate the plant tissues [27,28,46].

*Fus3/Kss1*-type MAPK homologs have been studied using knockout mutants, highlighting the crucial role of these genes in the infection process of the following phytopathogenic, entomopathogenic, and mycoparasitic fungi: *Magnaporthe oryzae* (*Pmk1*), *Pyrenophora teres* (*Ptk1*), *Cochliobolus heterostrophus* (*Chk1*), *Colletotrichum lagenarium* (*Cmk1*), *Fusarium oxysporum* (*Fmk1*), *Beauveria bassiana* (*BbMpk1*), *Metarhizium acridum* (*MaMk1*), and *Trichoderma virens* (*TmkA*) [33,47,48,49,50,51,52,53].

*Pls1* tetraspanins in *B. cinerea*, *Magnaporthe grisea*, and *Colletotrichum lindemuthianum* play a key role in the appressorium-mediated penetration into the host plant, since *Pls1* null mutations result in appressoria that are unable to form functional penetration pegs [30,31,32]. The expression of this phenomenon was clearly observed in *B. cinerea* using a transcriptional fusion between the *PLS1* promoter and an *EGFP* reporter gene [32].

The overall results obtained are in agreement with what has been observed by other authors, even when using different fungi and investigation techniques. Both *Fus3/Kss1*-type MAPKs and *Pls1* tetraspanins have conserved virulence-related functions among taxonomically different fungal species, including *B. cinerea* [20,34,54,55].

A topical application of dsRNA molecules that trigger a gene knockdown is a flexible approach that does not require transgenic plants for RNAi-based protection against plant diseases. We identified three dsRNA molecules in *B. cinerea*: *BcBmp3-*dsRNA [15], *BcBmp1-*dsRNA, and *BcPls1-*dsRNA (this work), which all showed a high efficacy in RNAi against the corresponding genes by the application of the exogenous dsRNA molecules produced in vitro. These dsRNAs are specific for *B. cinerea* and are related to functions involved in the pathogenicity/virulence of the fungus; they have been used for SIGS experiments on whole lettuce plants with very promising results [56].

However, this interesting biotechnological approach needs to overcome some aspects before being translated into practical applications.

The dsRNA molecules are susceptible to degradation when exposed to the environment, such as on the surface of plants or fruits. One of the best approaches to increase the stability and longevity of naked dsRNAs for topical applications is to complex them with biocompatible nanoparticles [40,57,58]. In tomatoes and chickpeas, Niño-Sánchez et al. [59] reported that dsRNAs carried by layered double hydroxide particles increased the effectiveness of the protection over time against *B. cinerea*.

SIGS-based disease management strategies require systems that can rapidly produce large quantities of dsRNA molecules and are cost-effective. Classical strategies for dsRNA production based on chemical synthesis or in vitro transcription are not feasible on a large scale due to high costs and low yields. The most suitable alternative is the production of dsRNAs using bacteria and yeasts as biofactories. In recent years, this option has allowed production costs to be significantly lowered, making the RNAi technique competitive on the market. Moreover, the products obtained from this technology are currently the subject of in-depth studies to verify their safety for the environment and for the consumer. Technical advances in the production and formulation of dsRNAs to improve their efficacy, stability, and persistence could therefore make it realistic to consider their use as “RNAi-based biofungicides” of a high commercial interest [8,9,60].

Looking ahead, RNAi can be considered a promising strategy due to its potential for the environmentally friendly control of *B. cinerea* as well as other economically important plant pathogenic fungi.

## 4. Materials and Methods

### 4.1. Fungal Strains and Culture Conditions

*Botrytis cinerea* B05.10 was the fungal pathogen used in this study. It was a haploid strain obtained after a benomyl treatment of the wild-type isolate SAS56 [61,62]. In this study, *Trichoderma harzianum* T6776 and *Fusarium oxysporum* DAFE SP-21-23 were used as off-target organisms. The *T. harzianum* isolate T6776 is known as a promising biocontrol agent against different plant pathogens [63], and the *F. oxysporum* isolate DAFE SP-21-23 was recovered from asymptomatic *Salicornia europea* plants.

All fungi were incubated on PDA (Biolife Italiana S.r.l., Milano, Italy) plates at 25 °C with a 12/12 NUV/light cycle unless indicated otherwise. The conidial suspensions were prepared from 7-to-10-day-old PDA cultures by gently scraping conidia from the surface of the culture with a sterile spatula in Sabouraud maltose broth (SMB: myco_logical peptone (Sigma-Aldrich, Saint Louis, MO, USA), 10 g L^−1^; maltose (Sigma-Aldrich, Saint Louis, MO, USA), 40 g L^−1^; pH, 5.6 ± 0.2) prepared with MilliQ water (EASYpure^®^ II LF, Thermo Scientific, Waltham, MA, USA; resistivity, 18.2 MΩ cm^−1^).

The resulting conidial suspension was filtered through a layer of sterile Miracloth (Calbiochem, San Diego, CA, USA), and the conidia concentration was checked with a hemacytometer (Bürker, LO—Laboroptik Ltd., Lancing, UK) and adjusted to the desired concentration with SMB.

### 4.2. In Vitro Effects of BcBmp1-dsRNA and BcPls1-dsRNAs on Growth and Conidial Germination of Botrytis cinerea

Fungal growth was studied in 96-well polystyrene microtiter plates (Cellstar^®^, Greiner Bio-One, Frickenhausen, Germany) to evaluate the effects of *BcBmp1*- and *BcPls1*-derived dsRNA (*BcBmp1*-dsRNA and *BcPls1*-dsRNA). Aliquots of a *B. cinerea* conidial suspension in SMB (5 × 10^2^ spores) and 2 µg (final concentration 20 ng µL^−1^) of *BcBmp1*- or *BcPls1*-dsRNA were added to the wells (*n* = 8 for each treatment in a total assay volume of 100 µL).

The controls included in each plate were prepared in SMB and consisted of: (i) 2 µg of *Green Fluorescent Protein* (*GFP*)*-*derived dsRNA (*GFP*-dsRNA) + *B. cinerea* conidia, (ii) no dsRNA + *B. cinerea* conidia + TE buffer (10 µM Tris/1.0 µM EDTA, pH of 7.0), and (iii) no dsRNA and no conidia + TE buffer (blank background control). The volume of TE corresponded exactly to the volume of the dsRNA added for the treatment.

The plates were incubated at 25 °C with a 12/12 NUV/light cycle and fungal growth was assessed by measuring the optical density (OD) at 595 nm with a microplate reader spectrophotometer (Bio-Rad, Model 680, Bio-Rad Laboratories, Cressier, Switzerland) at different times between 0 and 96 h. The experiments were repeated three times.

Absorbance values were converted to the percentage of fungal growth relative to that of the untreated control (100%) using the following formula:

[(OD_595_Control − OD_595_Treated)/OD_595_Control)] × 100

After 48 and 96 h of incubation, *B. cinerea* mycelium was collected and washed with sterile MilliQ water by centrifugation for a fungal transcript analysis to assess the silencing of the *BcBmp1* and *BcPls1* genes.

To determine the effect of *BcBmp1*- and *BcPls1*-derived dsRNA (*BcBmp1*-dsRNA and *BcPls1*-dsRNA) on the conidial germination of *B. cinerea*, the experiments were performed as previously described except, in this case, the conidial suspension was adjusted to 1 × 10^6^ conidia for each well. After 3, 6, and 9 h of incubation, germination rates were determined microscopically (Dialux 22, Leitz, Oberkochen, Germany) by taking small aliquots from each well. Conidia (*n* ≥ 200 for each biological replicate) were considered germinated when the germ tube length exceeded the conidial diameter. The percentage of germination was estimated by counting the number of germinated conidia relative to the total number of conidia. The experiment was performed in triplicate and repeated twice.

### 4.3. Botrytis cinerea Artificial Inoculations

*Lactuca sativa* cv. “Romana” (Romana Verde degli Ortolani, Sementi Dom Dotto S.p.A., Udine, Italy) plants were grown in a climate chamber with a 12 h photoperiod at 22 ± 1 °C with 65% relative humidity. Then, 20 µL of *BcBmp1*- or *BcPls1*-dsRNA (20 ng µL^−1^), *GFP*-dsRNA (20 ng µL^−1^), or sterile MilliQ water + TE buffer (10 µM Tris/1.0 µM EDTA, pH 7.0) were dropped on the adaxial surface of detached lettuce leaves (third or fourth pair) taken from 3-to-4-week-old plants (*n* = 16). The volume of TE corresponded exactly to the volume of the dsRNA added for the treatment. The dsRNAs were allowed to dry, and then 5 µL droplets of the *B. cinerea* conidial suspension (5 × 10^2^ spores) were placed on the same spot. The inoculated lettuce leaves were placed on sterile filter paper moistened with sterile MilliQ water in transparent plastic boxes (16 cm × 11.5 cm × 5 cm) and were incubated at 25 °C with a 12/12 NUV/light cycle. Infection symptoms were observed and photographed at 5 days post-inoculation (dpi). The necrotic areas (mm^2^) were measured using the ImageJ software, version 1.53a (http://imagej.nih.gov/ij, accessed on 21 October 2022) from digital images of the detached leaves [64]. The experiment was repeated twice.

### 4.4. DNA Extraction

For DNA extraction, the fungal mycelium of *B. cinerea* B05.10 was grown in sterile 50 mL Falcon tubes filled with 25 mL of SMB at 150 rpm (Rosi1000™, Thermolyne, Dubuque, IA, USA) for 2–4 d at 25 ± 1 °C. The mycelium was harvested by filtration through a layer of sterile Miracloth, washed with sterile MilliQ water, and dried on sterile filter paper. The total genomic DNA was extracted by the Genesig Easy DNA/RNA extraction kit (Primer Design Ltd., Chandler’s Ford, UK). The mycelium (200 mg) was placed into a 2 mL sterile extraction tube, which was prefilled with 0.5 mm of silica acid-washed glass beads (Sigma-Aldrich, Saint Louis, MO, USA) and 500 µL of sample prep solution supplied by the kit. The mycelium was then homogenized by a bead-beating method using the BeadBug™ Microtube homogenizer (Benchmark Scientific Inc., Sayreville, NJ, USA). Tubes were subjected to three beating cycles of 30 s at 4000 rpm, followed by 30 s on ice. The lysed suspension (200 µL) was collected and DNA extraction was performed by following the kit manufacturer’s instructions.

### 4.5. Sequence Analysis and Identification of BcBmp1 and BcPls1 Templates

We used the si-Fi software for the in silico analysis (siRNA Finder ver. siFi21_1.2.3-0008, https://sourceforge.net/projects/sifi21/, accessed on 7 February 2022), which is designed for RNAi silencing efficiency predictions and off-target analyses. This analysis was performed on the sequences of both the *BcBmp1* and *BcPls1* CDS of *B. cinerea* B05.10 (GenBank accession numbers NC_037311.1 and NC_037318.1, respectively; Figure S1A) in order to choose the optimal sequence for the design of dsRNAs to be employed in RNAi experiments (Figure S1B). The default parameters were used and the cDNA of *B. cinerea* B05.10 obtained from EnsemblFungi (http://ensemblgenomes.org/pub/fungi/release-50/fasta/botrytis_cinerea/cdna/ accessed on 7 February 2022) was selected as the database.

The genomic DNA of *B. cinerea* B05.10 was used as the template for the synthesis of a partial sequence of the *Mitogen-Activated Protein Kinase* (*MAPK*) *BcBmp1* gene (GenBank accession number NC_037311.1) that was 344 bp long (Table S3), and of a partial sequence of the *tetraspanin BcPls1* gene (GenBank accession number NC_037318.1) that was 413 bp long (Table S3). The PCR conditions were the following: 95 °C for 5 min; 5 cycles of 30 s at 95 °C, 30 s at 63 °C, and 30 s at 72 °C; 30 cycles of 30 s at 95 °C, 30 s at 77 °C, and 30 s at 72 °C; and 72 °C for 5 min.

The pCT74-sGFP plasmid [65] was used as the template for the synthesis of the *Green Fluorescent Protein* (*GFP*)-dsRNA (Table S3) of 712 bp. The PCR conditions were the following: 95 °C for 5 min; 5 cycles of 30 s at 95 °C, 30 s at 55 °C, and 30 s at 72 °C; 30 cycles of 30 s at 95 °C, 30 s at 76 °C, and 30 s at 72 °C; and 72 °C for 5 min. The DreamTaq DNA polymerase was used (Thermo Fischer Scientific, Vilnius, Lithuania).

The amplified products were purified and sequenced on both strands. The following software programs were used to analyze the amplified sequence: GENESCAN, FASTA, BLAST, and CLUSTALW, which are available at the National Center for Biotechnology Information (NCBI; http://www.ncbi.nlm.nih.gov/ accessed on 15 March 2022) [66]. Moreover, the PROSITE and PFAM databases were used to identify conserved domains [67,68]. The conserved motifs were also recognized by searching the conserved domain database (CDD) at NCBI and by the program InterPro at EMBL-EBI (http://www.ebi.ac.uk/ accessed on 20 March 2022). The analyses were performed using the representative genome of *B. cinerea* B05.10 [22,69] and the *GFP* sequence of the pCT74-sGFP plasmid [65].

### 4.6. dsRNA Synthesis

The dsRNAs were generated using the MEGAscript RNAi Kit (Invitrogen by Thermo Fisher Scientific, Vilnius, Lithuania) as previously described [15]. Primer pairs for *BcBmp1*-dsRNA, *BcPls1*-dsRNA (Figure S1A,B), and *GFP*-dsRNA with a T7 promoter sequence at the 5′end of both the forward and reverse primers were designed for the amplification of dsRNA (Table S3).

### 4.7. Gene Expression Analysis by Quantitative Real-Time Polymerase Chain Reaction (qRT-PCR)

The total RNA was extracted from 100 mg of mycelium; lettuce leaves treated with *GFP*-dsRNA, *BcBmp1*-dsRNA, or *BcPls1*-dsRNA; or untreated leaves (Ctrl). The extraction was performed using the RNeasy Plant Mini Kit (Qiagen, Milan, Italy) by following the manufacturer’s instructions. The concentration of each RNA sample was measured using the Qubit^TM^ RNA BR Assay Kit in a Qubit™ 4 Fluorometer (Invitrogen by Thermo Fisher Scientific Inc., Eugene, OR, USA), and the integrity was evaluated by agarose gel electrophoresis. The RNA samples were treated with amplification-grade DNase I (Sigma-Aldrich, St. Louis, MO, USA) and reverse-transcribed into cDNA (400 ng per sample) using the iScript cDNA synthesis kit (BioRad, Hercules, CA, USA). The synthesized cDNAs were used for quantitative Real-Time polymerase chain reaction (qRT-PCR) using gene-specific primer pairs (Table S3). qRT-PCRs were performed using Real-Time Step One apparatus (Applied Biosystem, Foster City, CA, USA) by using the recommended thermal-cycling conditions.

For the in vitro growth of *B. cinerea* mycelium, *BcSac7* (GenBank accession number XM_024693149.1) and *β-tubulin A* (*BctubA*, GenBank accession number XM_024690731.1) were selected as housekeeping genes. Although both the endogenous control genes tested exhibited stable expression among the different samples, *BctubA* was chosen to normalize the gene expression data for its high transcriptional stability.

For the inoculated lettuce leaves, genes encoding the TIP41-like protein (*LsTIP41*, GenBank accession number NC_056630.1), ubiquitin-NEDD8-like protein RUB2 (*LsUBQ-RUB2*, GenBank accession number NC_056624.1), and glyceraldehyde-3-phosphate dehydrogenase GAPC1, cytosolic (*LsGAPDH*, GenBank accession number NC_056630.1) were selected as housekeeping genes [70]. Although all the tested endogenous control genes exhibited a stable expression among the different samples, *LsGAPDH* was chosen to normalize the gene expression data for its high transcriptional stability. The amplifications of the target genes and the endogenous controls were run using three biological replicates, each with three technical replicates, and were analyzed on the same plate in separate tubes. The relative abundance of transcripts was calculated by using the 2^−∆∆*C*^_T_ method [71]. Relative transcript values were calculated using the Ctrl and *GFP* controls as reference samples. Before the quantification, a validation experiment was performed to ensure that the amplification efficiency of the target and reference genes was closely the same.

### 4.8. Off-Target Prediction

The precursor sequences used for the *BcBmp1*-dsRNA and *BcPls1*-dsRNA molecules were targeted against the complementary DNA (cDNA) databases of some phytopathogenic and beneficial fungi (*Sclerotinia sclerotiorum*, *Alternaria alternata*, *Fusarium oxysporum*, *Rhizoctonia solani*, *Pythium ultimum*, *Trichoderma asperellum*, *T. harzianum*, and *Rhizophagous irregularis*), humans, and lettuce using the si-Fi v21 software (default parameters) for the off-target prediction. The databases of three isolates of *B. cinerea*, including B05.10, were used as controls (Tables S1 and S2).

### 4.9. In Vitro Effects of BcBmp1- and BcPls1-Derived dsRNAs on Growth of the Off-Target Fungi Trichoderma harzianum T6776 and Fusarium oxysporum DAFE SP21-23

The effects of *Bmp1*- and *BcPls1*-derived dsRNAs (*BcBmp1*-dsRNA and *BcPls1*-dsRNA) on the fungal growth of *Trichoderma harzianum* T6776 and *Fusarium oxysporum* DAFE SP21-23 were investigated in 96-well polystyrene microtiter plates as described above for *B. cinerea* (Section 4.2). The optical density (OD of the fungal mycelium at different times (24, 48, 72, and 96 h) was measured at 595 nm.

### 4.10. Statistical Analysis

The data obtained from the in vitro assay in the 96-microtiter plates were converted as growth percentages of the untreated control. All data were subjected to an analysis of variance (ANOVA) using the statistical program CoStat 6.4 (Cohort Software, Monterey, CA, USA). The percentage data were transformed into arcsine √ % before the ANOVA. In the expression analysis, the values were the means (±SE) from three different biological replicates for each treatment. All the means were separated by Tukey’s honestly significant difference post-hoc (HSD) test (*p* ≤ 0.05). The normality of the data was tested using a Shapiro–Wilk test, whilst the homoscedasticity was tested using Bartlett’s test.

## Data Availability

The data are contained within the article or in the Supplementary Materials.

## Supplementary Materials

The following supporting information can be downloaded at: https://www.mdpi.com/article/10.3390/ijms24054869/s1.

## Abbreviations

*BcBmp1*	*Botrytis cinerea FUS3/KSS1-type Mitogen-Activated Protein Kinase* gene
*BcBmp3*	*Botrytis cinerea Slt2-type Mitogen-Activated Protein Kinase* gene
*BcPls1*	*Botrytis cinerea tetraspanin* gene
CDD	conserved domain database
CDS	coding sequence
Ctrl	control
dpi	days post-inoculation
dsRNA	double-stranded RNA
*GFP*	*Green Fluorescent Protein* gene
*hpRNA*	hairpin RNA
*LsGAPDH*	*Lactuca sativa Glyceraldehyde-3-Phosphate Dehydrogenase* protein gene
*LsTIP41*	*Lactuca sativa TIP41-like* protein gene
MAPKs	Mitogen-Activated Protein Kinases
NCBI	National Center for Biotechnology Information
non-GMO	non-Genetically Modified Organism
OD	optical density
PDA	Potato Dextrose Agar
PTGS	post-transcriptional gene silencing
qRT-PCR	quantitative Real-Time PCR
RNAi	RNA interference
SIGS	spray-induced gene silencing
siRNA	small interfering RNA
SMB	Sabouraud Maltose Broth
TE	Tris-EDTA buffer
