# Supplementary material for: Knockdown of Bmp1 and Pls1 Virulence Genes by Exogenous Application of RNAi-Inducing dsRNA in Botrytis cinerea
Source: Int J Mol Sci. 2023 Mar 2;24(5):4869. doi: 10.3390/ijms24054869 (PMC10003348; doi:10.3390/ijms24054869)
Supplement: Supplementary file 1 [file ijms-24-04869-s001.zip › ijms-2055611-supplementary.pdf]

International Journal of
Molecular Sciences

MDPI

Supplementary Materials

Knockdown of *Bmp1* and *Pls1* Virulence Genes by Exogenous Application of RNAi-Inducing dsRNA in *Botrytis cinerea*

Maria Spada[1], Claudio Pugliesi[1], Marco Fambrini[1], Diego Palpacelli[1], Susanna Pecchia[1,2,*]

[1] Department of Agriculture Food and Environment, University of Pisa, Via del Borghetto 80, 56124 Pisa, Italy;

[2] Interdepartmental Research Center Nutrafood "Nutraceuticals and Food for Health", University of Pisa, Via del Borghetto 80, 56124 Pisa, Italy

* Correspondence: susanna.pecchia@unipi.it

A)

ATGACAGCTCGTGCGCCTAATCCCGCCTCGGGATCACGGATCTCTTTCAATGTCAGCGACAATATGATATCCAGGATGTTGTAGGAG
AAGGAGCTTATGGTGTTGTCTGGTATGCTACCCCACTTTGTTACTCTTGCAATCTCTCGAATGGAGGCAGACCATGGAACTTGCAGA
AAATGGAGATGTGCTAATTCGATCTCCCGCTGTAGCTCAGCTTTGCACAAGCCTTCGGGGCAAAAGGTCGCTATCAAGAAGATTACT
CCATTCGATCACTCTATGTTCTGTTTGCGAACACTCCGTGAGATGAAGCTACTCCGATACTTCAATCACGAAAACATCATCTCCATT
CTCGACATCCAGAAGCCAAGAAACTATGAGTCCTTCACAGAAGTTTACCTTATCCAGGTATGTAGATTAGCAGCGTCAGGTTGAAAT
GGGATAATGTACTAATGCAAGAGCAGGAACTCATGGAGACCGATATGCATCGTGTAATTCGCACCCAAGATTTATCCGACGACCATT
GCCAATACTTTATCTATCAAACCCTGCGAGCCTTGAAAGCCATGCACTCCGCAAATGTTCTACACAGAGATTTGAAACCATCTAATC
TGTTACTCAATGCCAACTGCGATTTGAAGGTCTGCGATTTTGGTCTTGCTAGATCGGCTGCATCCCAAGAAGATAACTCTGGTTTCA
TGACAGAATATGTTGCGACCAGATGGTACCGTGCTCCAGAGATTATGTTGACGTTCAAGGAGTACACCAAGGCTATTGATGTCTGGT
CTGTTGGATGTATCTTGGCCGAGATGTTAAGCGGAAAGCCCCTGTTCCCTGGAAAGGATTGTGAGTGACGTCTGTATTGAAGTGATA
TCCCTTGCTAATCATTCATAGATCACCACCAACTCACACTCATTCTTGACGTTCTTGGTACTCCAACCATGGAAGATTACTACGGTA
TCAAATCTCGTCGTGCAAGAGAATATATCCGATCGTTGCCATTCAAGAAGAAGGTTCCTTTCAAGACCATGTTCCCAAAGACCTCAG
ATCTCGCTTTGGACCTCCTCGAGAAGCTTCTCGCTTTTAACCCAGTCAAGAGAATCACTGTCGAAGAAGCACTGAAGCACCCATACC
TTGAACCATACCACGATCCCGAAGATGAGCCAACCGCCAATCCCATTCCAGAAGAATTCTTTGATTTCGATAAAAACAAGGACAACC
TCACCAAGGAACAACTCAAAAAATTGATTTATGATGAGATCATGAGA**TAG**

B)

ATGGCGGATAAAATTCTTTTGACATATGTTATTCTGGACATACTCTTTGTAGGTTCAGGTGCATTATTGCTTGGGTTTGCCTTGACG
ACGAAGACTGGTACTTCGCAGGCTCCTACTATTGCCAGTGTAGCTACCGATTTGTTGTTGATGGGTACACCTTTGAATGGTTGGTTT
TCCCGTCTATTCATTATATTGTTGGTCTGGGAAGAAAGTATAGCAATGAGCTAACATCACTCGCACCCTATAGCCGCCATTGGAAAT
GCAATCTTGATCTTCTTCGCCTTCCTCATCTCCATTCCCGCCATGCTCCTCAGTACCACTCGCGGCTGGCTCAAACTTCATGGCTTC
TTCGTCGTTGTCTGTGGTCTCTTCACTCTCGTTATCGGTCTTGATATTTGGTTCGGTACACTAGAATCTAAACAATCTCTTCTCGAT
ACATGGATTGCCCAATCTGCGACCACGCAAAGTTTGTTGCAGGAACAATTAAGCTGTTGTGGATATTTCAATAGTACGAGTGCGCCA
GCTTTCGTAATCGATAGTACTTGTCCTAATGCTATTATTGCGGCCACAATGCCGGGATGTTCAGCGGCATTTGTTAAGTTGGATGGA
TTGTTCTTGGATGTCATCTTTACAGCGGCTTTTGGAATCGTGGGCTTGGATGTTGCGCTTATTTTTGGTATTGCAATGCTTAACAAG
GATCGTAAGGAGAGGGAAAGATATAGATTTATTGATGAGAAGAATGGAACGGGGTCTTTT**TAG**

Figure S1. (A) Nucleotide sequences of the gene *BcBmp1* of *Botrytis cinerea* B05.10 [22,69]. The start and stop codons are in bold characters and highlighted in green and magenta, respectively. The non-coding intronic regions are highlighted in yellow (GeneBank accession number NC_037311.1). The annealing part of the primers with T7 promoter are highlighted in light blue and gray (see also Table S2). **(B)** Nucleotide sequences of the gene *BcPls1* of *Botrytis cinerea* B05.10 [22,69]. The start and stop codons are in bold characters and highlighted in green and magenta, respectively. The non-coding intronic region is highlighted in yellow (GeneBank accession number NC_037318.1). The annealing part of the primers with T7 promoter are highlighted in light blue and gray (see also Table S3).

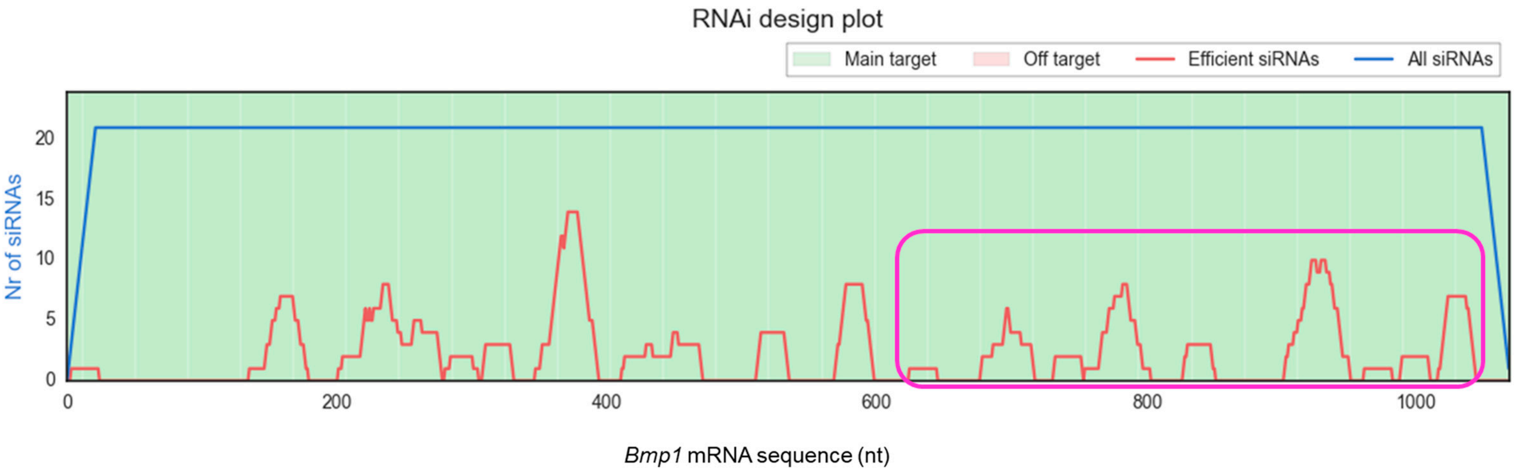

Figure S2. Graphical output of the predicted total and efficient siRNA hits in the *Botrytis cinerea* B05.10 *Bmp1* mRNA calculated by the si-Fi v21 software. The pink box includes the sequence used to generate the fragment of 344 bp for the synthesis of dsRNA (fourth exon of the gene, see Fig. S1A).

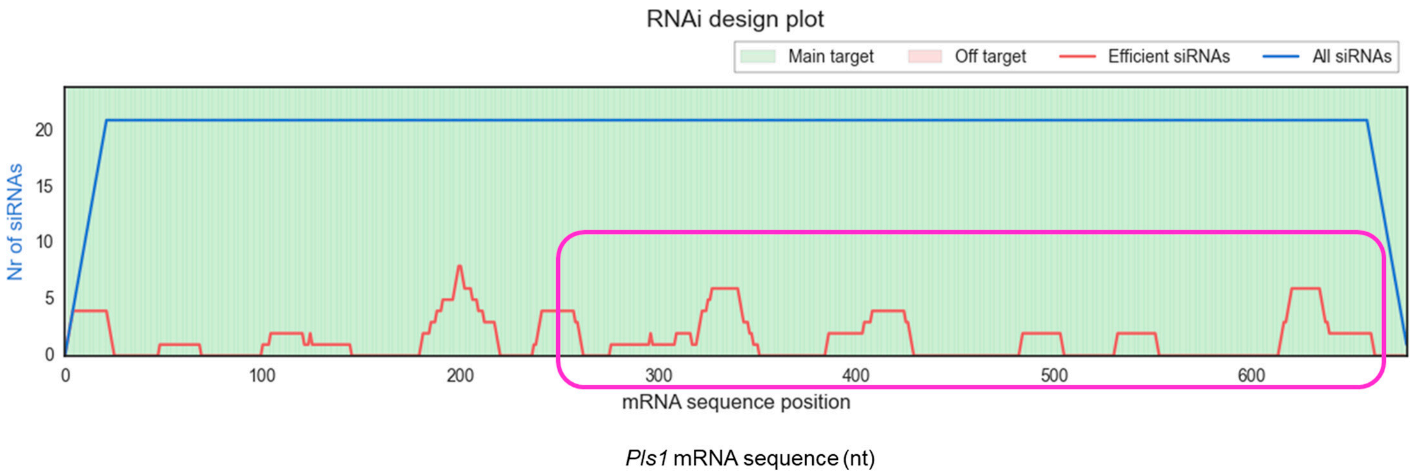

Figure S3. Graphical output of the predicted total and efficient siRNA hits in the *Botrytis cinerea* B05.10 *Pls1* mRNA calculated by the si-Fi v21 software. The pink box includes the sequence used to generate the fragment of 413 bp for the synthesis of dsRNA (second exon of the gene; see Figure S1B).

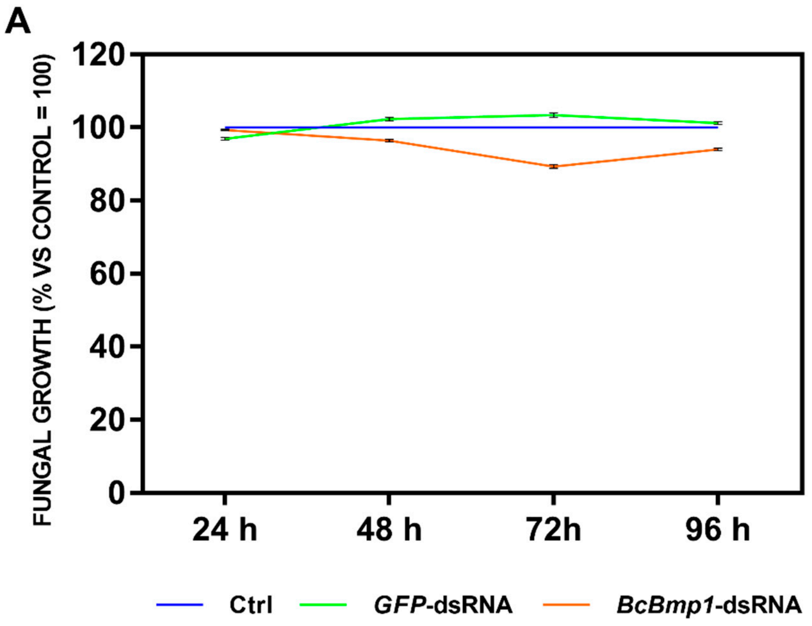

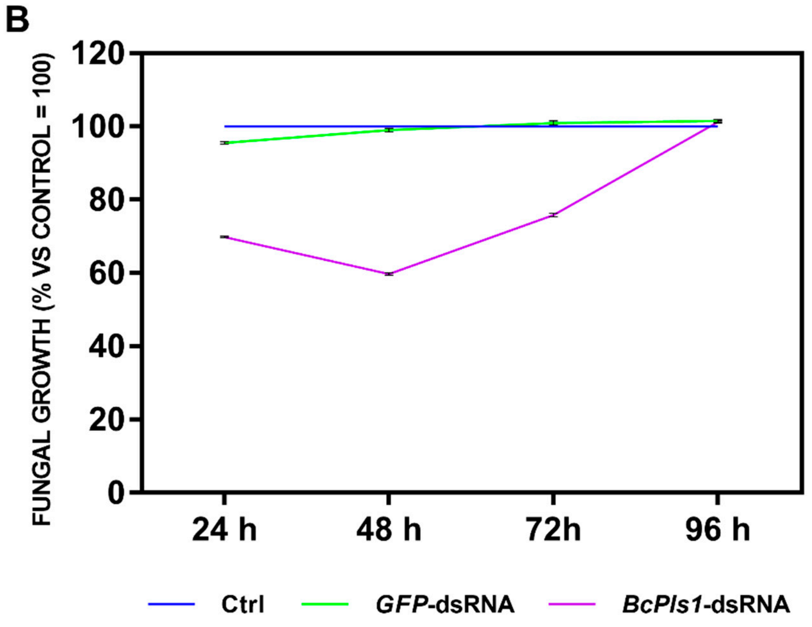

Figure S4. In vitro effects of *BcBmp1*-dsRNA **(A)** and of *BcPls1*-dsRNA **(B)** on *B. cinerea* B05.10 growth. Fungal growth was assessed measuring the optical density (OD) at 595 nm at 24, 48, 72 and 96 hours (24 h, 48 h, 72 h and 96 h) in 96-well microtiter plates. In each well there are aliquots of a conidial suspension of *B. cinerea* B05.10 (5×10^2 spores) in SMB medium and 2 µg of dsRNA (*GFP*-dsRNA (green) or *BcBmp1*-dsRNA (orange), or *BcPls1*-dsRNA (purple). SMB + TE buffer was used as control (Ctrl, blue). The graphs show the mean (±SE) of three independent experiments with eight biological replicates (*n* = 8). Data obtained from the in vitro assay were converted as growth percentage of the untreated control.

Table S1. Prediction of *BcBmp1* off-target transcripts using the si-Fi v21 software.

Copyright: © 2023 by the authors. Licensee MDPI, Basel, Switzerland. This article is an open access article distributed under the terms and conditions of the Creative Commons Attribution (CC BY) license (https://creativecommons.org/licenses/by/4.0/).

	All siRNA[*]	Efficient siRNA[**]
Botrytis cinerea B05.10[a]	324	158
Botrytis cinerea T4[b]	324	158
Botrytis cinerea DW1[c]	324	158
Sclerotinia sclerotiorum[d]	0	0
Sclerotinia sclerotiorum 1980 UF-70[e]	78	37
Alternaria alternata SRC1lrK2f[f]	0	0
Fusarium oxysporum[g]	0	0
Rhizoctonia solani AG-1 IA[h]	0	0
Pythium ultimum ASM14694[i]	0	0
Trichoderma asperellum CBS 433.97[l]	0	0
Trichoderma harzianum T6776[m]	0	0
Rhizophagous irregularis DAOM 197198[n]	0	0
Lactuca sativa cv. Salinas[o]	0	0
Homo sapiens (GRCh38.p13)[p]	0	0

Below the links of the cDNA gene sequence files are reported:

[a] ftp://ftp.ensemblgenomes.org/pub/fungi/release-50/fasta/botrytis_cinerea/cdna/

[b] ftp://ftp.ensemblgenomes.org/pub/fungi/release-50/fasta/fungi_ascomycota1_collection/botrytis_cinerea_t4_gca_000227075/cdna/

[c] ftp://ftp.ensemblgenomes.org/pub/fungi/release-50/fasta/fungi_ascomycota1_collection/botrytis_cinerea_bcdw1_gca_000349525/cdna/

[d] ftp://ftp.ensemblgenomes.org/pub/fungi/release-50/fasta/sclerotinia_sclerotiorum/cdna/

[e] ftp://ftp.ensemblgenomes.org/pub/fungi/release-50/fasta/fungi_ascomycota3_collection/sclerotinia_sclerotiorum_1980_uf_70_gca_001857865/cdna/

[f] ftp://ftp.ensemblgenomes.org/pub/fungi/release-50/fasta/fungi_ascomycota3_collection/alternaria_alternata_gca_001642055/cdna/

[g] ftp://ftp.ensemblgenomes.org/pub/fungi/release-50/fasta/fusarium_oxysporum/cdna/

[h] ftp://ftp.ensemblgenomes.org/pub/fungi/release-50/fasta/fungi_basidiomycota1_collection/rhizoctonia_solani_ag_1_ia_gca_000334115/cdna/

[i] ftp://ftp.ensemblgenomes.org/pub/protists/release-50/fasta/pythium_ultimum/cdna/

[l] ftp://ftp.ensemblgenomes.org/pub/fungi/release-50/fasta/fungi_ascomycota4_collection/trichoderma_asperellum_cbs_433_97_gca_003025105/cdna/

[m] ftp://ftp.ensemblgenomes.org/pub/fungi/release-50/fasta/fungi_ascomycota2_collection/trichoderma_harzianum_gca_000988865/cdna/

[n] ftp://ftp.ensemblgenomes.org/pub/fungi/release-50/fasta/fungi_mucoromycota1_collection/rhizophagus_irregularis_daom_197198w_gca_000597685/cdna/

[o] https://www.ncbi.nlm.nih.gov/assembly/GCA_002870075.2

[p] http://ftp.ensembl.org/pub/release-103/fasta/homo_sapiens/cdna/

[*] Number of siRNA sequences (21-mer) that perfectly match the query sequence.

[**] Number of siRNA sequences (21-mer) with perfect match to the query sequence that meet additional criteria for efficient RNAi.

Table S2. Prediction of *BcPls1* off-target transcripts using the si-Fi v21 software.

	All siRNA*	Efficient siRNA**
Botrytis cinerea B05.10[a]	393	193
Botrytis cinerea T4[b]	308	152
Botrytis cinerea DW1[c]	393	193
Sclerotinia sclerotiorum[d]	7	4
Sclerotinia sclerotiorum 1980 UF-70[e]	7	4
Alternaria alternata SRC1lrK2f[f]	0	0
Fusarium oxysporum[g]	0	0
Rhizoctonia solani AG-1 IA[h]	0	0
Pythium ultimum ASM14694[i]	0	0
Trichoderma asperellum CBS 433.97[l]	0	0
Trichoderma harzianum T6776[m]	0	0
Rhizophagous irregularis DAOM 197198[n]	0	0
Lactuca sativa cv. Salinas[o]	0	0
Homo sapiens (GRCh38.p13)[p]	0	0

Below the links of the cDNA gene sequence files are reported:

[a] ftp://ftp.ensemblgenomes.org/pub/fungi/release-50/fasta/botrytis_cinerea/cdna/

[b] ftp://ftp.ensemblgenomes.org/pub/fungi/release-50/fasta/fungi_ascomycota1_collection/botrytis_cinerea_t4_gca_000227075/cdna/

[c] ftp://ftp.ensemblgenomes.org/pub/fungi/release-50/fasta/fungi_ascomycota1_collection/botrytis_cinerea_bcdw1_gca_000349525/cdna/

[d] ftp://ftp.ensemblgenomes.org/pub/fungi/release-50/fasta/sclerotinia_sclerotiorum/cdna/

[e] ftp://ftp.ensemblgenomes.org/pub/fungi/release-50/fasta/fungi_ascomycota3_collection/sclerotinia_sclerotiorum_1980_uf_70_gca_001857865/cdna/

[f] ftp://ftp.ensemblgenomes.org/pub/fungi/release-50/fasta/fungi_ascomycota3_collection/alternaria_alternata_gca_001642055/cdna/

[g] ftp://ftp.ensemblgenomes.org/pub/fungi/release-50/fasta/fusarium_oxysporum/cdna/

[h] ftp://ftp.ensemblgenomes.org/pub/fungi/release-50/fasta/fungi_basidiomycota1_collection/rhizoctonia_solani_ag_1_ia_gca_000334115/cdna/

[i] ftp://ftp.ensemblgenomes.org/pub/protists/release-50/fasta/pythium_ultimum/cdna/

[l] ftp://ftp.ensemblgenomes.org/pub/fungi/release-50/fasta/fungi_ascomycota4_collection/trichoderma_asperellum_cbs_433_97_gca_003025105/cdna/

[m] ftp://ftp.ensemblgenomes.org/pub/fungi/release-50/fasta/fungi_ascomycota2_collection/trichoderma_harzianum_gca_000988865/cdna/

[n] ftp://ftp.ensemblgenomes.org/pub/fungi/release-50/fasta/fungi_mucoromycota1_collection/rhizophagus_irregularis_daom_197198w_gca_000597685/cdna/

[o] https://www.ncbi.nlm.nih.gov/assembly/GCA_002870075.2

[p] http://ftp.ensembl.org/pub/release-103/fasta/homo_sapiens/cdna/

* Number of siRNA sequences (21-mer) that perfectly match the query sequence.

** Number of siRNA sequences (21-mer) with perfect match to the query sequence that meet additional criteria for efficient RNAi.

Target	Total siRNA hits	Efficient siRNA hits
BcBmp1	324	158

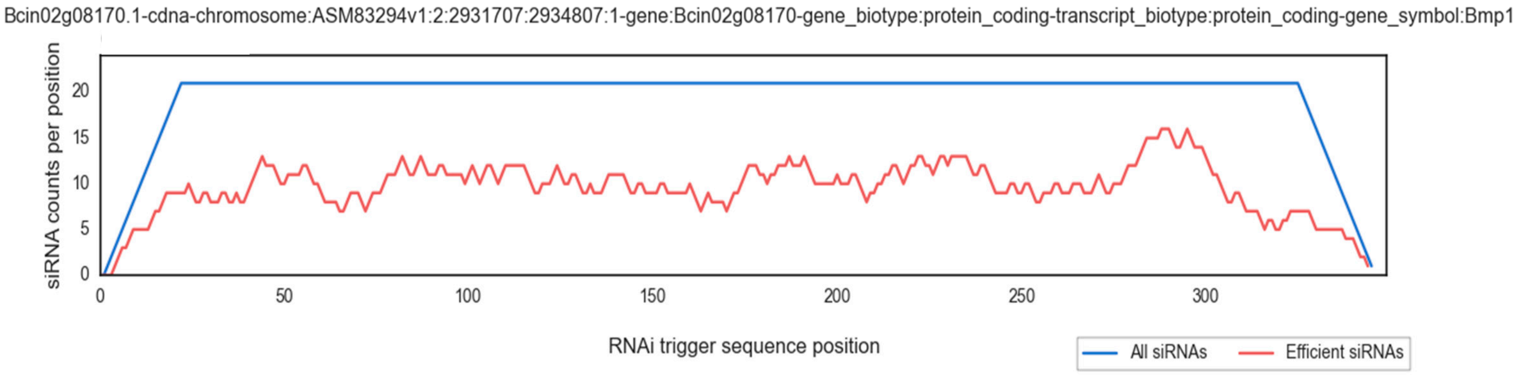

Figure S5. Graphical output of the predicted total and efficient siRNA hits in the *BcBmp1* fragment used for the synthesis of dsRNA calculated by the si-Fi v21 software.

Target	Total siRNA hits	Efficient siRNA hits
BcPls1	393	193

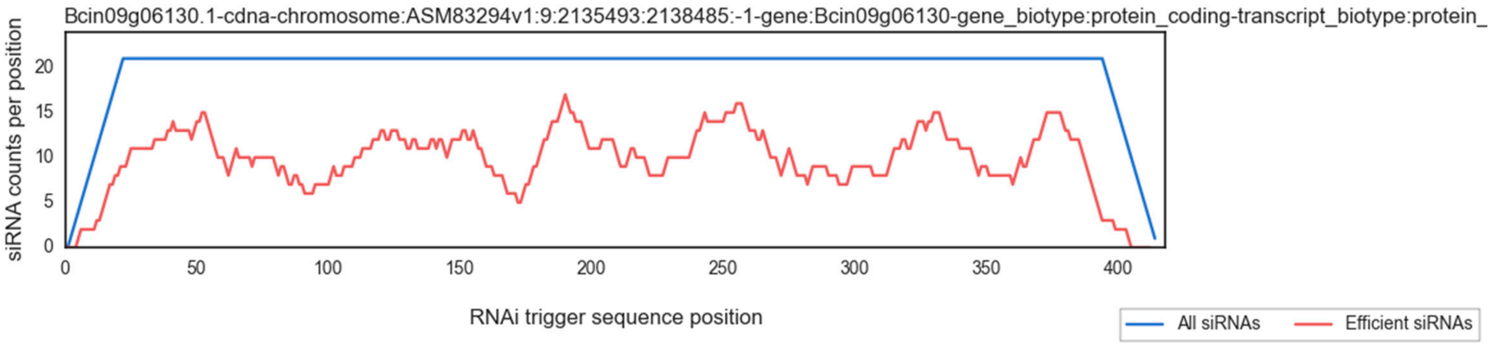

Figure S6. Graphical output of the predicted total and efficient siRNA hits in the *BcPls1* fragment used for the synthesis of dsRNA calculated by the si-Fi v21 software.

Table S3. Gene-specific primers used in this study. In square brackets is indicate the chromosome number of *Botrytis cinerea* B05.10 [22,69] and *Lactuca sativa* [70].

**Use**	**GenBank accession number**	**Primer**	**Primer sequence 5′-3′ (F: Forward; R: Reverse)**	**Amplicon size (bp)**
Amplification of the *Bmp1* gene fragment from *Botrytis cinerea* (*BcBmp1*) [BCIN02] for *in vitro* transcription	NC_037311.1	BcBmp1T7F<br>BcBmp1T7R	F:TAATACGACTCACTATAGGGAGAATCACCACCAACTCACACTCAT<br>R:TAATACGACTCACTATAGGGAGAGAGTTGTTCCTTGGTGAGGTTG	344
Amplification of the *Pls1* gene fragment from *Botrytis cinerea* (*BcPls1*) [BCIN09] for *in vitro* transcription	NC_037318.1	BcPls1T7F<br>BcPls1T7R	F:TAATACGACTCACTATAGGGAGACATGGCTTCTTCGTCGTTGT<br>R:TAATACGACTCACTATAGGGAGAGACCCCGTTCCATTCTTCTC	413
Amplification of the *GFP* gene fragment from plasmid pCT74-sGFP for *in vitro* transcription		GFP1T7F<br>GFP1T7R	F:TAATACGACTCACTATAGGGAGAGTGAGCAAGGGCGAG<br>R:TAATACGACTCACTATAGGGAGATTGTACAGCTCGTCCAT	713
Amplification by qRT-PCR of the *Bmp1* gene of *Botrytis cinerea* (*BcBmp1*) [BCIN02]	NC_037311.1	qBmp1F<br>qBmp1R	F:CTATCAAACCCTGCGAGCCT<br>R:CTGGTCGCAACATATTCTGTCA	183
Amplification by qRT-PCR of the *Pls1* gene of *Botrytis cinerea* (*BcPls1*) [BCIN09]	NC_037318.1	qPls1F<br>qPls1R	F:CGCCTTCCTCATCTCCATTC<br>R:CAACGACGAAGAAGCCATGAA	80

Amplification by qRT-PCR of the *GAPDHR1* gene of *Lactuca sativa* (*LsGAPDHR1*) [LS8]	NC_056630.1	LsGAPDHF<br>LsGAPDHR	F:CTTCAACATCATTCCCAGCAG<br>R:GCCTTCTTCTCAAGTCTAACAGT	150
Amplification by qRT-PCR of the *UBQ-RUB2* gene of *Lactuca sativa* (*LsUBQ-RUB2*) [LS2]	NC_056624.1	LsUBQ7F<br>LsUBQ7R	F:GCAGCGACACCATCGACAAT<br>R:CAGCGAGTGTTCTTCCATCTT	116
Amplification by qRT-PCR of the *TIP41* gene of *Lactuca sativa* (*LsTIP41*) [LS5]	NC_056627.1	LsTIP41F<br>LsTIP41R	F:CATTCGAGAGATTTGCTGGAG<br>R:TGACTGATGATGTTTGGATCGTTG	102
Amplification by qRT-PCR of the housekeeping gene *beta tubulin A* of *Botrytis cinerea* (*BctubA*) [BCIN01]	XM_024690731.1	qBtubF<br>qBtubR	F:GTCTCAAGATGTCCTCCACC<br>R:ACTCCATCTCGTCCATACCT	144
Amplification by qRT-PCR of the housekeeping gene *Sac7* of *Botrytis cinerea* (*BcSac7*) [BCIN05]	XM_024693149.1	qSac7F<br>qSac7R	F:CTCAGTGGCTCGGAAAAGC<br>R:CGTTTGCCGCATCATGAACTG	112

Disclaimer/Publisher's Note: The statements, opinions and data contained in all publications are solely those of the individual author(s) and contributor(s) and not of MDPI and/or the editor(s). MDPI and/or the editor(s) disclaim responsibility for any injury to people or property resulting from any ideas, methods, instructions or products referred to in the content.
